# Clinical Relevance of NGAL/MMP-9 Pathway in Patients with Endometrial Cancer

**DOI:** 10.1155/2017/6589262

**Published:** 2017-09-27

**Authors:** Aneta Cymbaluk-Płoska, Anita Chudecka-Głaz, Ewa Pius-Sadowska, Agnieszka Sompolska-Rzechuła, Karolina Chudecka, Michał Bulsa, Bogusław Machaliński, Janusz Menkiszak

**Affiliations:** ^1^Department of Gynecological Surgery and Gynecological Oncology of Adults and Adolescents, Pomeranian Medical University, Al. Powstańców Wielkopolskich 72, 70-111 Szczecin, Poland; ^2^General Pathology Department, Pomeranian Medical University, Al. Powstańców Wielkopolskich 72, 70-111 Szczecin, Poland; ^3^Department of Mathematics Applications in Economy, West Pomeranian University of Technology, al. Piastów 17, 70-310 Szczecin, Poland

## Abstract

The objectives of the study were to assess the relationship between the serum levels of MMP-9 and NGAL and the clinical staging and histopathological grade of the tumor. Lipocalin-2/NGAL and MMP-9 concentrations were quantified in serum by multiplex fluorescent bead-based immunoassays (Luminex Corporation, Austin, TX, USA). The AUC values for NGAL and MMP-9 were 0.9 and 0.78, respectively. The diagnostic potential of NGAL and MMP-9 in differentiating high-stage (FIGO III and IV) and low-stage (FIGO I and II) cancer and predicting the cell differentiation grade (G1 versus G3) on the basis of the analyses of AUC values was determined to be 0.91 and 0.79 for NGAL and 0.82 and 0.84 for MMP-9, respectively. Multifactorial logistic regression analysis in the final method revealed that NGAL and MMP-9 variables were independent of the endometrial cancer risk. OR values for NGAL and MMP-9 were 1.23 (95% CI 1.421–3.27; *p* = 0.034) and 1.09 (95% CI: 1.38–4.12; *p* = 0.026), respectively. The NGAL/MMP-9 complex may be useful in the assessment of tumor stage before surgical treatment.

## 1. Introduction

Endometrial cancer is one of the most common types of tumors. According to Globocan data, about 320,000 new cases of endometrial cancer were recorded in 2012 [1]. The incidence rates in developed countries cannot be reduced. This is associated largely to the prevalence or endometrial cancer risk factors such as obesity, hypertension, and diabetes [2]. The 5-year survival rate in treated patients with low-stage endometrial cancer is about 80%. Patients with high risk of recurrence pose a significant challenge for researchers. Due to the high prevalence of the disease, the search for clinically useful prognostic and predictive factors is under way. To date, no single protein was found that could be considered an independent prognostic factor in endometrial cancer. Recent publications reported high expression of metalloproteinases in endometrial tumors. Metalloproteinases are a group of extracellular endopeptidases involved in the degradation of extracellular matrix. The main role in the metalloproteinase family is played by MMP-9 also referred to as gelatinase B. MMP-9 is an extracellular enzyme degrading the extracellular matrix and the basal membrane [3]. MMP-9 is also a lipocalin-2-associated endopeptidase. Lipocalin-2 is a protein comprising a risk factor of insulin resistance and diabetes [4, 5]. Higher levels of lipocalin-2 were observed in obese patients as well as in patients with elevated levels of estrogens and glucocorticoids in blood serum [6]. The role of lipocalin-2/MMP-9 complex consists in prevention of MMP-9 degradation and thus in the strengthening of the role of MMP-9 as a factor involved in tumor progression [7, 8].

## 2. Objectives

The objectives of the study are the following: to examine the behavior of MMP-9 and NGAL levels in patients with endometrial cancer and benign endometrial disorders and to assess the relationship between the serum levels of MMP-9 and NGAL and the clinical staging and histopathological grade of the tumor.

## 3. Material

The study included 143 patients who were admitted due to vaginal bleeding. All patients signed informed consent to participate in the study. The study protocol was approved by the Pomeranian Medical University Ethical Committee. Following surgical treatment, the results of histopathological examination patients were assorted into four groups:

1. Patients with endometrial cancer (*n* = 80)
2. Patients with normal endometrium (*n* = 23)
3. Patients with endometrial polyps (*n* = 20)
4. Patients with endometrial leiomyomas (*n* = 20)

Among the group of patients with endometrial cancers, we identified 67 patients with endometrial cancer, 9 patients with endometrial cancer with serous component, and 3 patient was diagnosed with adenocarcinoma with partial squamous differentiation and one with adenocarcinoma with part of clear cell carcinoma.

Patients from the endometrial cancer group were divided according to tumor grading into G1 = 23, G2 = 36, and G3 = 21 subgroups, as well as depending on clinical tumor staging:

1. FIGO I and II patients, *n* = 61
2. FIGO III and IV patients, *n* = 19

Variables analyzed in the study included the depth of myometrial infiltration, vascular and lymphatic system invasion, and the status of lymph node metastases.  Table 1 presents the detailed distribution of individual subgroups of the population.

Five millilitres of blood was collected from each patient for the determination of lipocalin-2, MMP-9 levels on the occasion of routine preoperative testing, and centrifugation. The serum was subsequently frozen and stored at −70°C.

## 4. Methods

### 4.1. Assay Analysis MMP-9

25 *μ*L of each standard, control, and samples was added to the plate together with multiplex antibody capture bead solution, and the plate was incubated with agitation for 2 h at room temperature. Subsequently, the well was washed with 200 *μ*L wash buffer 2 times by using a hand-held magnet. 25 *μ*L of detection antibody cocktail was pipette to each well, and the plate was sealed and incubated at room temperature for 1 hour on a plate shaker. After this step, 25 *μ*L streptavidin-phycoerythrin mixture was added to the plate and incubated with agitation for 30 minutes in the dark. Finally, after washing, the microspheres in each well were resuspended in 100 *μ*L sheath fluid and shaken at room temperature for 5 minutes. The plate was then read and analyzed on the Luminex analyzer, and analyte concentrations were determinated from five different standard curves showing MFI (median fluorescence intensity) versus protein concentration.

Lipocalin-2/NGAL concentrations were quantified in serum by multiplex fluorescent bead-based immunoassays (Luminex Corporation, Austin, TX, USA) using commercial human cardiovascular disease (CVD) Magnetic Bead Panel 2 (Merck Millipore, Billerica, MA, USA). 25 *μ*L of each standard, control, and samples was added to the plate together with multiplex antibody capture bead solution, and the plate was incubated with agitation overnight at 40°C. Subsequently, the well was washed with 200 *μ*L wash buffer 3 times by using a hand-held magnet. 50 *μ*L of detection antibody cocktail was pipette to each well, and the plate was sealed and incubated at room temperature for 1 hour on a plate shaker. After this step, 50 *μ*L streptavidin-phycoerythrin mixture was added to the plate and incubated with agitation for 30 minutes in the dark. Finally, after washing, the microspheres in each well were resuspended in 150 *μ*L sheath fluid and shaken at room temperature for 5 minutes. The plate was then read and analyzed on the Luminex analyzer, and analyte concentrations were determinated from five different standard curves showing MFI (median fluorescence intensity) versus protein concentration.

The statistical analysis was performed using STASTICA 10.0 PL program. The descriptive characteristic of the examined population of patients was prepared, determining minimum, maximum mean, and median values. Because the distributions of the analyzed features to compare mean values are not normal distributions, median positional parameters and nonparametric tests were used (the Kruskal-Wallis test and post hoc Dunn test in the comparison of three groups and Mann–Whitney *U* test in the comparison of two groups). For the selected groups, the receiver operating characteristic (ROC) curves were obtained and the area under curve (AUC) was calculated with 95% confidence intervals according to the nonparametric method of DeLong. A *p* value of <0.05 was considered statistically significant. The study variables were analyzed using the logistic regression model.

The model facilitates the examination of the impacts of multiple independent variables on a binary dependent variable *Y*. The values of variable *Y* are coded as follows: 1: presence of a particular trait and 0: absence of a particular trait. The function used in the description of the logistic regression follows an extended S-shaped curve. Logistic regression coefficients may be determined using the maximum likelihood method or the generalized least squares method. Due to the nonlinearity of the model in relation to the independent variables and parameters, the logistic model is transformed into the linear regression model using logarithmic transformation. To this end, the concept of odds ratio (OR) is introduced as the ratio between the likelihood of a particular event and the likelihood of that event not happening. Therefore, the odds ratio is used to express the factor of the increase or the decrease in the likelihood of a particular event upon a unit change in the independent variable (with fixed values of the remaining independent variables).

## 5. Results

### 5.1. Comparative Analysis of the Study Groups

Table 2 presents the mean serum levels of individual proteins.

**Figure 1:**
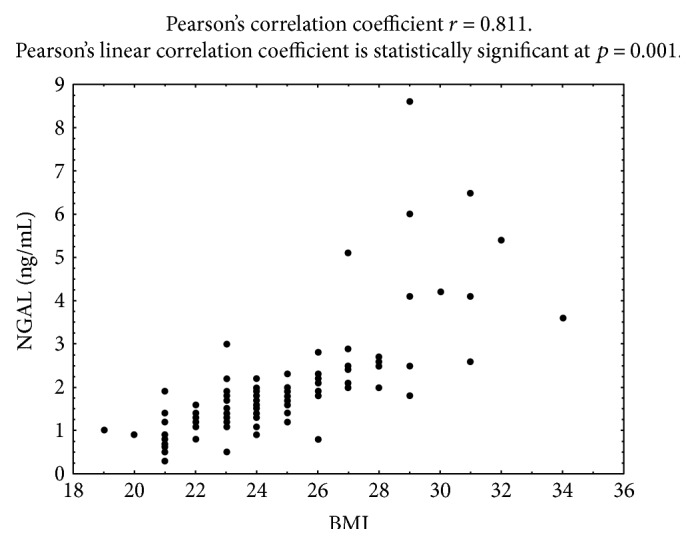
Correlation between mean concentrations of NGAL and BMI in the whole study patient group.

Statistically significant correlations were observed between the patients' BMI values and lipocalin-2 levels (*r* = 0.82, *p* = 0.001) in the entire study population (Figure 1). No analogous correlations were observed between the BMI values or blood glucose levels and the serum levels of MMP-9. Blood glucose levels in patients with type 2 diabetes were shown to be correlated with serum NGAL levels (*r* = 0.64); no correlation was observed between the NGAL levels and the blood pressure measurements. No correlation could also be observed between the blood pressure measurements and the serum levels of MMP-9 (Table 3). The analysis of NGAL levels in patients with endometrial cancer as compared to patients with normal endometrium and to patients with endometrial polyps revealed statistically significant differences between the compared groups corresponding to *p* = 0.002 for the normal endometrium group and *p* = 0.004 for the endometrial polyp group. Differences were also observed in MMP-9 levels between the patients with endometrial cancer and patients with normal endometrium (*p* = 0.001) or endometrial polyps (*p* = 0.003).

In the comparison of mean protein levels between the patients with endometrial cancer and the patients with endometrial myomas, the statistical significance of differences was stronger for MMP-9 as compared to NGAL (*p* = 0.0002/*p* = 0.005). The analysis of NGAL/MMP-9 indicator in patients with endometrial cancer as compared to patients with benign endometrium changes was statistically significant differences between the compared groups—64.7 pg/mL/44.4 pg/mL (*p* = 0.001).

### 5.2. Comparative Analysis of Protein Levels in Relation to Prognostic Factors

Following the analysis of all the hitherto recognized unfavorable prognostic factors of endometrial cancer, we determined that the NGAL levels were higher in patients with higher stage of endometrial cancer (FIGO III and IV versus FIGO I and II) (*p* = 0.001) as well as in patients with poorly differentiated G3 tumors as compared to patients with well-differentiated G1 tumors (*p* = 0.003). In cases of blood and lymphatic vessel invasion and the presence of lymph node metastases, serum NGAL levels were higher when lymph nodes were involved (*p* = 0.002; *p* = 0.003; and *p* = 0.001) (Table 4).

Serum MMP-9 levels were statistically significantly higher in high-stage tumors (*p* = 0.001), in poorly differentiated tumors (*p* = 0.02), in cases of blood vessel invasion (*p* = 0.004), in proportion to the depth of myometrial infiltration (*p* = 0.01), and upon lymph node involvement (*p* = 0.003). The results are presented in Table 5.

The statistically significant differences for the NGAL/MMP-9 indicator were found between the mean concentrations in patients according to staging and grading. Significantly higher indicator was in patients with high-stage and poor histopathological differentiation. It was 83.3 pg/mL/61.2 pg/mL (*p* = 0.004) and 77.5 pg/mL/62.2 pg/mL (*p* = 0.03), respectively.

### 5.3. Analysis of ROC Curves

**Figure 2:**
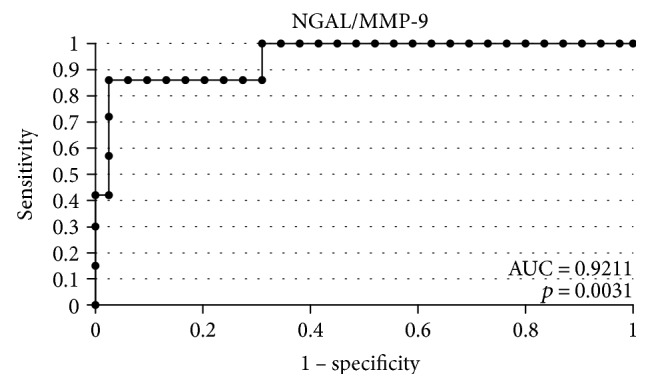
The ROC curves for indicator NGAL/MMP-9 in women. The analysis compared endometrial cancer patients to patients with benign endometrial lesions.

**Figure 3:**
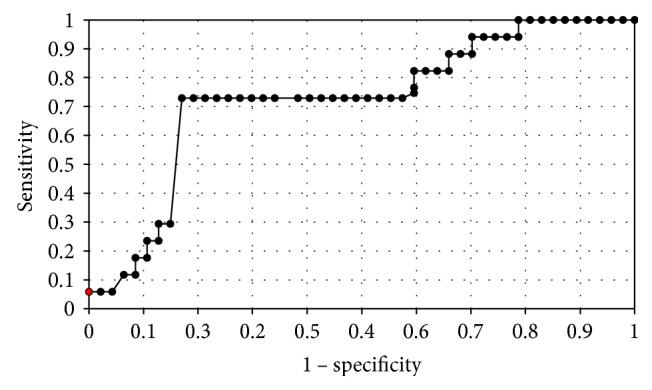
The ROC curves for MMP-9 proteins in women. The analysis compared endometrial cancer patients to patients with benign endometrial lesions.

**Figure 4:**
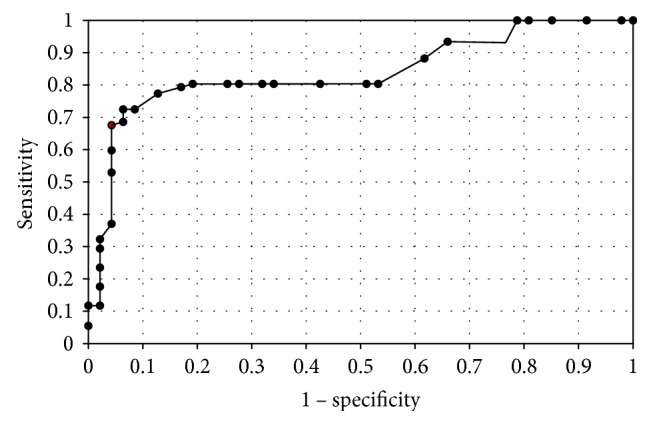
The ROC curves for NGAL proteins in women. The analysis compared endometrial cancer patients to patients with benign endometrial lesions.

**Figure 5:**
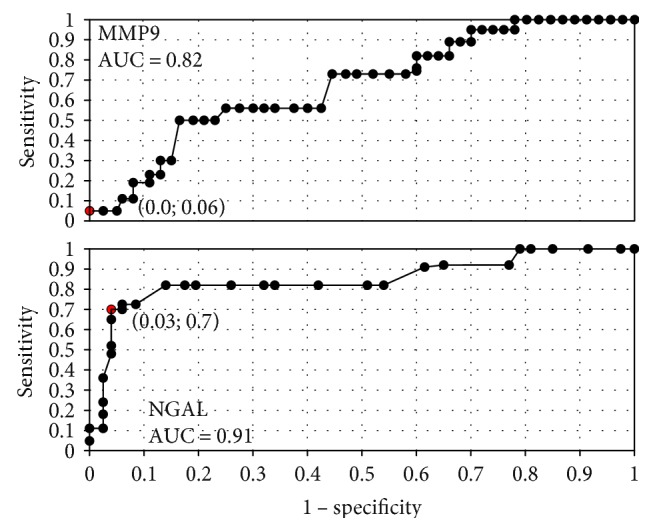
The ROC curves for MMP-9 and NGAL proteins depending on staging.

**Figure 6:**
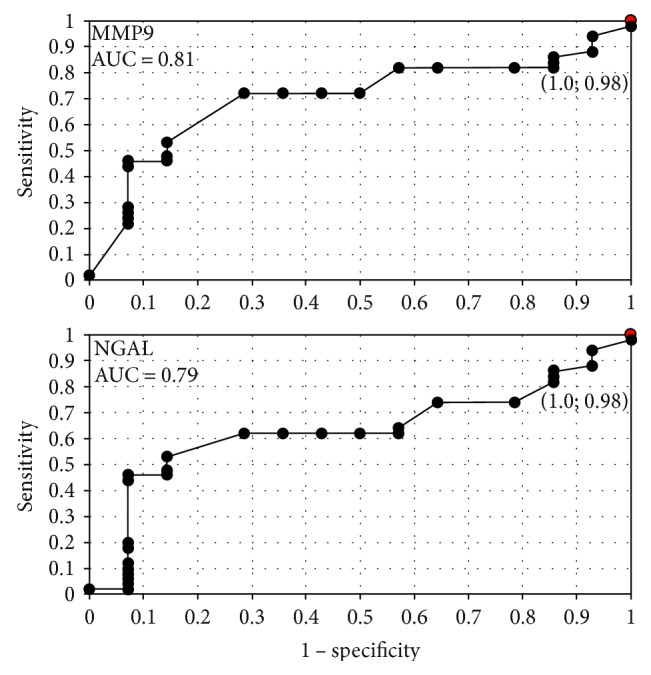
The ROC curves for MMP-9 and NGAL proteins in G1 and G3 grading.

**Figure 7:**
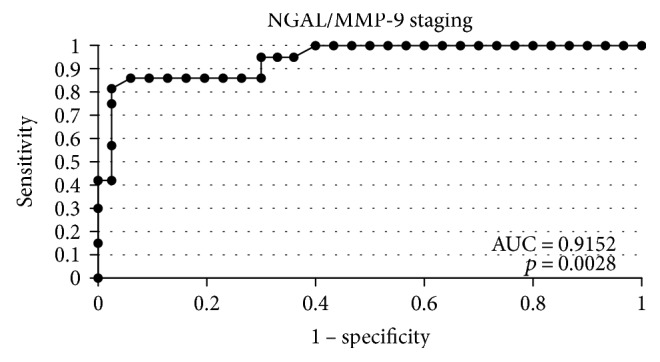
The ROC curves for indicator NGAL/MMP-9 depending on staging.

**Figure 8:**
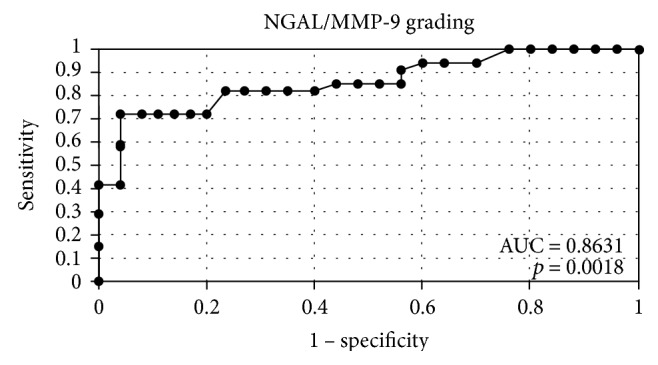
The ROC curves for indicator NGAL/MMP-9 depending on grading.

In order to evaluate the diagnostic value of lipocalin-2 and MMP-9, the ROC curves were plotted and the areas under the ROC curves (AUC) were calculated. The analysis compared the patients with endometrial cancer to patients with benign endometrial lesions. The AUC values for NGAL and MMP-9 were 0.9 and 0.78, respectively. The AUC value for NGAL/MMP-9 indicator which compared the patients with endometrial cancer to patients with benign endometrial changes was 0.92 (Figure 2). The diagnostic potential of NGAL, MMP-9, and indicator NGAL/MMP-9 in differentiating high-stage (FIGO III and IV) and low-stage (FIGO I and II) cancer and predicting the cell differentiation grade (G1 versus G3) on the basis of the analyses of AUC values was determined to be 0.91 and 0.79 for NGAL, 0.82 and 0.84 for MMP-9, and 0.91, 0.86 for indicator NGAL/MMP-9, respectively. The curves are presented in Figures 3, 4, 5, 6, 7, and 8. Four logistic regression models were performed. Multifactorial logistic regression analysis in the final method revealed that NGAL and MMP-9 variables were independent of the endometrial cancer risk. OR values for NGAL and MMP-9 were 1.23 (95% CI: 1.421–3.27; *p* = 0.034) and 1.09 (95% CI: 1.38–4.12; *p* = 0.026), respectively. In addition, the same independent variables were analyzed with regard to the risk of lymph node metastases (yes/no), tumor stage FIGO III and IV versus FIGO I and II, and grading G3 versus G1. The respective OR values for NGAL and MMP-9 were as follows: 1.4 (95% CI: 0.9–2.98; *p* = 0.02)/2.02 (95% CI: 3.1–6.42; *p* = 0.004); 3.66 (95% CI: 4.2–12.3; *p* = 0.005)/1.28 (95% CI: 1.58–7.2; *p* = 0.03); and 1.09 (95% CI:1.8–5.2; *p* = 0.03)/1.83 (95% CI: 2.4–8.9; *p* = 0.02) (Table 6).

## 6. Discussion

The main risk factors of type I endometrial cancer have been known for years and include obesity, diabetes, and arterial hypertension. The adipose tissue plays the role of a secretory organ releasing a number of adipokines that play an important role in the system. One of such released glycoproteins is NGAL, that is, lipocalin-2. It acts as an insulin resistance enhancer [9] and participates in the process of oncogenesis [10–12]. MMP-9 is a protein that plays an apparently crucial role in the proliferation of cells in endometrial cancer. It belongs to the family of endoproteinases with iron-, zinc-, and calcium-dependent activity. Metalloproteinases degrade the components of extracellular matrix removing the barrier between tumor cells and normal tissue environment, thus initiating the metastatic process. Numerous reports identified a complex of MMP-9, that is, gelatinase B with NGAL (neutrophil gelatinase-associated lipocalin), a low-molecular protein. By strengthening the bonds in MMP-9 molecules, NGAL protects them from autodegradation. As demonstrated in in vitro studies, both MMP-9 and NGAL led to the increase in tumor sizes [13, 14].

In our study, we were able to demonstrate that patients with endometrial cancer presented with NGAL and MMP-9 levels higher than those with healthy endometrium or benign endometrial lesions. As observed by Mannelqvist et al., NGAL expression was higher in endometrial cancer patients compared to cancer-free patients [15]. Reports of higher expression of MMP-9 in endometrial cancer patients were also published [16].

In their recent study from 2016, Li et al. highlighted that the high expression of NGAL can be considered to be significantly correlated with the expression of vimentin and the migration, invasion, and proliferation of tumor cells [11].

In our study, we were able to demonstrate statistically significant differences only with regard to the lipocalin-2 levels in well- and poorly differentiated tumors with no differences being observed between G1 and G2 tumors. On the other hand, Li et al. demonstrated that NGAL levels in G1 tumor patients are statistically significantly different from those in patients with healthy endometrium. Thus, one may expect that NGAL is a biomarker that may find its potential use in early detection of endometrial cancer. This, however, must be confirmed in future studies. In addition, a study in endometrial cancer cell lines revealed that an increase in NGAL expression is observed during epithelial-mesenchymal transformation [11].

We found that the NGAL levels were significantly higher in patients with higher tumor stages and were correlated with infiltration of lymphatic and blood vessels as well as lymph node metastases. Similar reports were presented by Mannelqvist et al. who, in their multifactorial analysis, identified NGAL as an independent prognostic factor of tumor grading and staging in endometrial cancer patients [15]. Lee et al. conducted a study in a transgenic mouse model to achieve inhibition of tumorigenesis following application of NGAL inhibitor [17].

The effect of inflammation on carcinogenesis in genital organ tumors is another issue to be considered. In their meta-analysis of 832 cases of endometrial cancer, Delahanty et al. observed that inflammation played an important role in the carcinogenesis of endometrial tumors [18]. Elevated expression of MMP-9 and IL-6 was also confirmed. The authors take note of the importance of NGAL as its levels are significantly increased in inflammatory processes [19, 20].

MMP-9 appears to play a crucial role in the formation of metastases of endometrial cancer. Increased MMP-9 expression is associated with myometrial invasion as well as with invasion into blood and lymphatic vessels [21]. Karahan et al. demonstrated that the increase in MMP-9 expression may be correlated to both tumor size and aggressiveness [16].

The increased expression of MMP-9 in endometrial cancer is correlated with the overexpression of other proteins, for example, with overexpression of Ki67 and tumor necrosis factor alpha-induced protein-8 [22], epidermal growth factor [23], and fibroblast growth factor [24], as well as with the increased activity of telomerase, and overexpression of catalytic protein hTERT [25, 26].

No correlation between MMP-9 expression and patient survival was presented by Fang Yu et al. who highlighted the significant impact of myometrial infiltration depth, blood and lymphatic vessel invasion, and lymph node metastases but not of the MMP-9 levels on the predicted survival in endometrial cancer patients.

Our study was the first one to assess the total serum levels of NGAL and MMP-9 using the logistic regression model which facilitates attempted estimation of the preoperative risk of endometrial cancer. We are looking for a new, easy method to perform research of new cancer markers, which will allow us to evaluate and assess tumor progression. In this paper, the analysis of individual proteins and then the index was calculated, but the NGAL/MMP-9 complex was not evaluated. Determination of individual proteins is cheaper method, and calculation of NGAL/MMP-9 indicator is not difficult. Research may be carried out with a small quantity of test material. This method is relatively new and innovative, using a combination of two techniques, ELISA and cytometry. In the future, we are planning to compare NGAL/MMP-9 sensitivity and specificity indicator and NGAL/MMP-9 complex, both in serum and in urine.

## 7. Conclusion

Both NGAL and MMP-9 proteins may be useful in assessing the stage of the cancer, before surgical treatment. Further studies are needed to be conducted to confirm the prognostic significance of the GAL/MMP-9 indicator.

## Figures and Tables

**Table 1:** Patients with endometrial cancer divided into subgroups.

Subgroups	Distribution	Numbers
The histopathological type	Type I cancer (endometrial endometrioid adenocarcinoma)	*n* = 67
	Type II cancer patients (serous endometrial carcinoma, squamous adenocarcinoma, and clear cell carcinoma)	*n* = 13
Histopathological grade of the tumor	G1	*n* = 23
	G2	*n* = 36
	G3	*n* = 21
Clinical stage of the tumor	FIGO I and II	*n* = 61
	FIGO III and IV	*n* = 19
Myometrial infiltration depth	Superficial myometrial infiltration (<1/2 of the thickness)	*n* = 53
	Deep myometrial infiltration (>1/2 of the thickness)	*n* = 27
Vascular space involvement	With vascular invasion	*n* = 36
	Without vascular invasion	*n* = 44
Lymph vessel involvement	With lymph vessel invasion	*n* = 27
	Without lymph vessel invasion	*n* = 53
Lymph node metastases	With lymph node metastases	*n* = 22
	Without lymph node metastases	*n* = 58

**Table 2:** Comparative analysis of the study groups.

Variable	*n*	Mean range	Median (95.000%–95.000%)	*n*	Mean range	Median (95.000%–95.000%)	*p*
	Carcinoma endometrium			Normal endometrium			
MMP-9 pg/mL	80	8089.6 (6481.1–8891.7)	8234.2 (6341.6–8732.1)	23	5698.2 (3987.1–6341.1)	5712.1 (4251.4–6652.3)	0.001
NGAL ng/ml	80	180 (120–240)	171 (130–231)	23	110 (90–134)	113 (106–127)	0.002
	Carcinoma endometrium			Polyp endometrium			
MMP-9 pg/ml	80	8089.6 (6481.1–8891.7)	8234.2 (6341.6–8732.1)	20	6213.3 (4235.1–7123.8)	6438.1 (4521.2–7234.8)	0.003
NGAL ng/ml	80	180 (120–240)	171 (130–231)	20	118 (107–150)	110 (99–142)	0.004
	Carcinoma endometrium			Myoma			
MMP-9 pg/ml	80	8089.6 (6481.1–8891.7)	8234.2 (6341.6–8732.1)	20	4923.1 (3615.2–6213.4)	5022.1 (3871.4–6431.1)	0.0002
NGAL ng/ml	80	180 (120–240)	171 (130–231)	20	132 (113–170)	128 (120–161)	0.005

**Table 3:** NGAL and MMP-9 concentration levels in patients with or without endometrial cancer risk factors.

	NGAL pg/ml	MMP-9 ng/ml	
Obesity	230 (170–254)	7234.5 (6345–8234)	Mean range
Without obesity	150 (110–188)	7465.6 (6234–8767)	
*p*	0.001	>0.05	
Diabetes t2	210 (148–243)	7632.3 (7142–8111)	Mean range
Without diabetes t2	132 (103–163)	7231.4 (6721–8082)	
*p*	0.002	>0.05	
Hypertension	189 (138–201)	6721.1 (6234.6–7341.3)	Mean range
Without hypertension	160 (131–192)	7561.4 (7098–8212)	
*p*	>0.05	>0.05	

**Table 4:** Comparative analysis according to the prognostic factors for NGAL.

	Mean	Median	Min	Max	*p*
Endometroid/non-endometroid	176/193	175/191	132/139	190/240	0.054
G1/G2	142/161	144/168	120/126	172/180	0.081
G1/G3	142/191	144/201	121/168	172/240	0.003
FIGO I and II/FIGO III and IV	140/220	142/226	123/178	179/280	0.001
Vascular invasion (±)	152/203	149/201	120/170	179/258	0.002
Lymph vessel invasion (±)	156/213	160/221	136/177	180/262	0.003
Lymph node metastasis (±)	155/231	157/230	131/180	179/281	0.001
Infiltrate the myometrium superficial/deep	158/185	156/186	140/149	182/232	0.066

**Table 5:** Comparative analysis according to the prognostic factors for MMP-9.

	Mean	Median	Min	Max	*p*
Endometroid/non-endometroid	7721.5/8909.8	7543.6/8823.3	7012.6/8002.3	8231/9567.6	0.04
G1/G2	5263/6789.2	5324.2/6881.2	4967.2/5122.5	5778.1/7657.7	0.061
G1/G3	5263/8782.1	5324.2/889.2	4967.2/6744.5	5778.1/989.0	0.02
FIGO I and II/FIGO III and IV	5433.1/9213.7	5367/9311.7	4977.2/7338.4	6877.2/10131.8	0.001
Vascular invasion (±)	5688.3/8672.9	5723.3/8721.5	5002.9/7112.4	5989.7/9881.7	0.004
Lymph vessels invasion (±)	6345.2/7187.2	6463.1/7132.2	4412.5/5323.9	7889.2/8999.3	0.07
Lymph nodes metastasis (±)	5998.1/9141.8	6021/9099.3	5121.9/7111	6781.9/10267	0.003
Infiltrate the myometrium superficial/deep	6891.3/9234.8	6631.4/9122.4	5899.6/7114.8	7346.8/10145.6	0.01

**Table 6:** Multifactorial logistic regression models for NGAL and MMP-9 for staging, grading, and lymph node metastasis.

Protein	OR	95% CI	*p*
	Lymph node metastasis		
NGAL	1.4	0.9–2.98	0.02
MMP-9	2.02	3.1–6.42	0.004
	FIGO III and IV versus I and II		
NGAL	3.66	4.2–12.3	0.005
MMP-9	1.28	1.58–7.2	0.03
	GRADING III versus I		
NGAL	1.09	1.8–5.2	0.03
MMP-9	1.83	2.4–8.9	0.02

## References

[1] Ferlay J., Soerjomataram I., Ervik M. GLOBOCAN 2012 v1.0. Cancer incidence and mortality worldwide: IARC CancerBase No. 11. http://globocan.iarc.fr/Pages/fact_sheets_cancer.aspx.

[2] Renehan A. G., Soerjomataram I. (2016). Obesity as an avoidable cause of cancer (attributable risks). *Recent Results in Cancer Research*.

[3] Yadav L., Puri N., Rastogi V., Satpute P., Ahmad R., Kaur G. (2014). Matrix metalloproteinases and cancer - roles in threat and therapy. *Asian Pacific Journal of Cancer Prevention*.

[4] Wang Y., Lam K. S., Kraegen E. W. (2007). Lipocalin-2 is an inflammatory marker closely associated with obesity, insulin resistance, and hyperglycemia in humans. *Clinical Chemistry*.

[5] Yan Q. W., Yang Q., Mody N. (2007). The adipokine lipocalin 2 is regulated by obesity and promotes insulin resistance. *Diabetes*.

[6] Kamble P. G., Pereira M. J., Sidibeh C. O. (2016). Lipocalin 2 produces insulin resistance and can be upregulated by glucocorticoids in human adipose tissue. *Molecular and Cellular Endocrinology*.

[7] Lin H. H., Liao C. J., Lee Y. C., Hu K. H., Meng H. W., Chu S. T. (2011). Lipocalin-2-induced cytokine production enhances endometrial carcinoma cell survival and migration. *International Journal of Biological Sciences*.

[8] Candido S., Di Maso M., Serraino D. (2016). Diagnostic value of neutrophil gelatinase-associated lipocalin/matrix metalloproteinase-9 pathway in transitional cell carcinoma of the bladder. *Tumour Biology*.

[9] Cabia B., Andrade S., Carreira M. C., Casanueva F. F., Crujeiras A. B. (2016). A role for novel adipose tissue-secreted factors in obesity-related carcinogenesis. *Obesity Reviews*.

[10] Liao C. J., Huang Y. H., Au H. K., Wang L. M., Chu S. T. (2012). The cancer marker neutrophil gelatinase-associated lipocalin is highly expressed in human endometrial hyperplasia. *Molecular Biology Reports*.

[11] Li T., Yu L., Wen J., Liao Q., Liu Z. (2016). An early-screening biomarker of endometrial carcinoma: NGAL is associated with epithelio-mesenchymal transition. *Oncotarget*.

[12] Marchewka Z., Tacik A., Piwowar A. (2016). KIM-1 and NGAL as potential biomarkers for the diagnosis and cancer progression. *Postȩpy Higieny i Medycyny Doświadczalnej (Online)*.

[13] Mira E., Lacalle R. A., Buesa J. M. (2004). Secreted MMP9 promotes angiogenesis more efficiently than constitutive active MMP9 bound to the tumor cell surface. *Journal of Cell Science*.

[14] Yang J., Moses M. A. (2009). Lipocalin 2: a multifaceted modulator of human cancer. *Cell Cycle*.

[15] Mannelqvist M., Stefansson I. M., Wik E. (2012). Lipocalin 2 expression is associated with aggressive features of endometrial cancer. *BMC Cancer*.

[16] Karahan N., Guney M., Baspinar S., Oral B., Kapucuoglu N., Mungan T. (2007). Expression of gelatinase (MMP-2 and MMP-9) and cyclooxygenase-2 (COX-2) in endometrial carcinoma. *European Journal of Gynaecological Oncology*.

[17] Lee Y. C., Tzeng W. F., Chiou T. J., Chu S. T. (2012). MicroRNA-138 suppresses neutrophil gelatinase-associated lipocalin expression and inhibits tumorigenicity. *PLoS One*.

[18] Delahanty R. J., Xiang Y. B., Spurdle A. (2013). Polymorphisms in inflammation pathway genes and endometrial cancer risk. *Cancer Epidemiology, Biomarkers & Prevention*.

[19] Zhang J., Wu Y., Zhang Y., Leroith D., Bernlohr D. A., Chen X. (2008). The role of lipocalin 2 in the regulation of inflammation in adipocytes and macrophages. *Molecular Endocrinology*.

[20] Kim S., Kim H. J., Ahn H. S. (2016). Is plasma neutrophil gelatinase-associated lipocalin a predictive biomarker for acute kidney injury in sepsis patients? A systematic review and meta-analysis. *Journal of Critical Care*.

[21] Di Nezza L. A., Misajon A., Zhang J. (2002). Presence of active gelatinases in endometrial carcinoma and correlation of matrix metalloproteinase expression with increasing tumor grade and invasion. *Cancer*.

[22] Liu T., Gao H., Yang M., Zhao T., Liu Y., Lou G. (2014). Correlation of TNFAIP8 overexpression with the proliferation, metastasis, and disease-free survival in endometrial cancer. *Tumour Biology*.

[23] Cho-Clark M., Larco D. O., Zahn B. R., Mani S. K., Wu T. J. (2015). GnRH-(1-5) activates matrix metallopeptidase-9 to release epidermal growth factor and promote cellular invasion. *Molecular and Cellular Endocrinology*.

[24] Xue S. N., Lei J., Yang C., Lin D. Z., Yan L. (2012). The biological behaviors of rat dermal fibroblasts can be inhibited by high levels of MMP9. *Experimental Diabetes Research*.

[25] Ding D., Xi P., Zhou J., Wang M., Cong Y. S. (2013). Human telomerase reverse transcriptase regulates MMP expression independently of telomerase activity via NF-*κ*B-dependent transcription. *The FASEB Journal*.

[26] Kong W., Lv N., Wysham W. Z. (2015). Knockdown of hTERT and treatment with BIBR1532 inhibit cell proliferation and invasion in endometrial cancer cells. *Journal of Cancer*.

